# A deep-learning automated image recognition method for measuring pore patterns in closely related bolivinids and calibration for quantitative nitrate paleo-reconstructions

**DOI:** 10.1038/s41598-023-46605-y

**Published:** 2023-11-10

**Authors:** Anjaly Govindankutty Menon, Catherine V. Davis, Dirk Nürnberg, Hidetaka Nomaki, Iines Salonen, Gerhard Schmiedl, Nicolaas Glock

**Affiliations:** 1 Department of Earth System Sciences, Institute for Geology, Universität Hamburg, Bundesstrasse 55, 20146 Hamburg, Germany; 2 Department of Marine, Earth, and Atmospheric Sciences, North Carolina State University, 2800 Faucette Dr, Raleigh, NC 27695 USA; 3 GEOMAR Helmholtz Centre for Ocean Research Kiel, Wischhofstr. 1-3, Geb. 8c, Raum 106, 24148 Kiel, Germany; 4 SUGAR, X-star, Japan Agency for Marine-Earth Science and Technology (JAMSTEC), 2-15 Natsushima-cho, Yokosuka, 237-0061 Japan; 5 Present Address: Tvärminne Zoological Station, Faculty of Biological and Environmental Sciences, University of Helsinki, Hanko, Finland; 6 Center for Earth System Research and Sustainability, Institute for Geology, Universität Hamburg, Bundesstrasse 55, 20146 Hamburg, Germany

**Keywords:** Ocean sciences, Marine biology, Biogeochemistry, Palaeoceanography

## Abstract

Eutrophication is accelerating the recent expansion of oxygen-depleted coastal marine environments. Several bolivinid foraminifera are abundant in these oxygen-depleted settings, and take up nitrate through the pores in their shells for denitrification. This makes their pore density a possible nitrate proxy. This study documents three aspects related to the porosity of bolivinids. 1. A new automated image analysis technique to determine the number of pores in bolivinids is tested. 2. The pore patterns of *Bolivina spissa* from five different ocean settings are analysed. The relationship between porosity, pore density and mean pore size significantly differs between the studied locations. Their porosity is mainly controlled by the size of the pores at the Gulf of Guayaquil (Peru), but by the number of pores at other studied locations. This might be related to the presence of a different cryptic *Bolivina* species in the Gulf of Guayaquil. 3. The pore densities of closely related bolivinids in core-top samples are calibrated as a bottom-water nitrate proxy. *Bolivina spissa* and *Bolivina subadvena* showed the same correlation between pore density and bottom-water nitrate concentrations, while the pore density of *Bolivina argentea* and *Bolivina subadvena accumeata* is much higher.

## Introduction

Oceanic oxygen concentrations are predicted to decrease globally affecting the stability of marine ecosystems^1–4^. Global warming accelerates ongoing ocean deoxygenation^5,6^, and expansion of oxygen minimum zones (OMZs)^1,2,7^. Increased ocean warming enhances upper-ocean stratification^8^, reduces ventilation, and has implications for biological productivity^7^ as well as carbon, nitrogen^9^ and phosphorus cycling^10^ in the oceans. These processes are amplified by the large-scale use of chemical nitrogenous fertilizers to satisfy global demand for food production which drastically disrupts the nitrogen cycle^11,12^. Oxygen is a major influence on the marine nitrogen cycle in the global oceans^6^ as some microbial processes require oxygen while others are inhibited by it^8^. When oxygen concentrations drop below ~ 4.5 µmol/kg, nitrate becomes the major electron acceptor for respiration replacing oxygen, a condition called suboxic^13–15^. The continued expansion of suboxia results in the loss of fixed nitrogen via denitrification^14,16^, a dissimilatory process in which nitrate (NO_3_^-^) is ultimately converted into dinitrogen gas^17^. Therefore, denitrification reduces the supply of NO_3_^-^ in global oceans^14,16^. Nitrogen fixation, nitrification, and denitrification are major processes in the nitrogen cycle that are mainly facilitated by bacteria^18^, while lower oxygen concentrations can either enhance or inhibit these processes^14^. Therefore, the nitrogen cycling in OMZs is different from the rest of the open ocean^15^. Approximately 30–50% of fixed nitrogen loss in the world’s oceans occurs in oxygen minimum and deficient zones^14^. Quantitative paleo-reconstruction of nitrate levels could provide a comprehensive understanding of how the different processes mentioned above interacted in the past. This will help us to predict future changes in marine nutrient budgets and possible impacts of eutrophication.

Foraminifera are a group of amoeboid protists that are abundant in marine environments^19^, and account for a major part of benthic denitrification in the OMZs^20–22^. Many calcareous foraminiferal tests (shells) are porous. The pores in benthic foraminiferal tests play an important role in facilitating gas exchange and osmoregulation between the foraminifera and the environment^23^. The pore density (number of pores per unit area), mean pore size (average pore sizes of one individual), and shape of pores are important morphological features that vary among different taxa^24–26^. The porosity (% of the area of the tests occupied by the pores), and pore density of foraminifera are likely driven by environmental factors. Factors that have been suggested include latitude, water density^27–30^, temperature, salinity^31^, oxygen, and nitrate concentrations^24,32–34^. Porosity might also be genetically encoded^25,35^. Porosity is a species-specific trait that can be used to distinguish certain pseudocryptic species such as *Ammonia* spp.^25^. Nevertheless, within a single species phenotypic plasticity exists. Thus, porosity can be influenced by environmental conditions, and hence used as a paleoproxy.

Porosity in benthic foraminifera plays an important role in adaptation strategies by facilitating gas exchange through larger pore areas in low oxic conditions^24,32,36^. Cell organelles involved in respiration (i.e. mitochondria) are more abundant around the inner pore surfaces of species living in oxygen-depleted conditions than in well-oxygenated conditions^23^. In some foraminiferal species, increased gas exchange can be attained by either increasing the number of pores or by increasing the surface area of the test (or shell)^37^. However, the function of pores may vary among species because of their difference in evolutionary history^38^.

The shallow oxygen minimum zones of the Eastern Pacific have large standing stocks of benthic foraminiferal species^39^. Several benthic foraminiferal species living in oxygen-depleted environments perform complete denitrification, which is rare amongst eukaryotes^40^. Denitrification is the preferred respiration pathway in several foraminiferal species from oxygen-depleted environments, making these eukaryotes an important part of benthic nitrogen cycling in some environments^41^. Previously, it has been found that benthic foraminifera living in oxygen- or nitrate-depleted environments have higher pore density and porosity than those living under well-oxygenated conditions or high ambient nitrate concentrations^24,32,33^. Therefore, pore parameters of fossil shells are promising proxies for paleo oxygen and nitrate concentrations.

**Figure 1:**
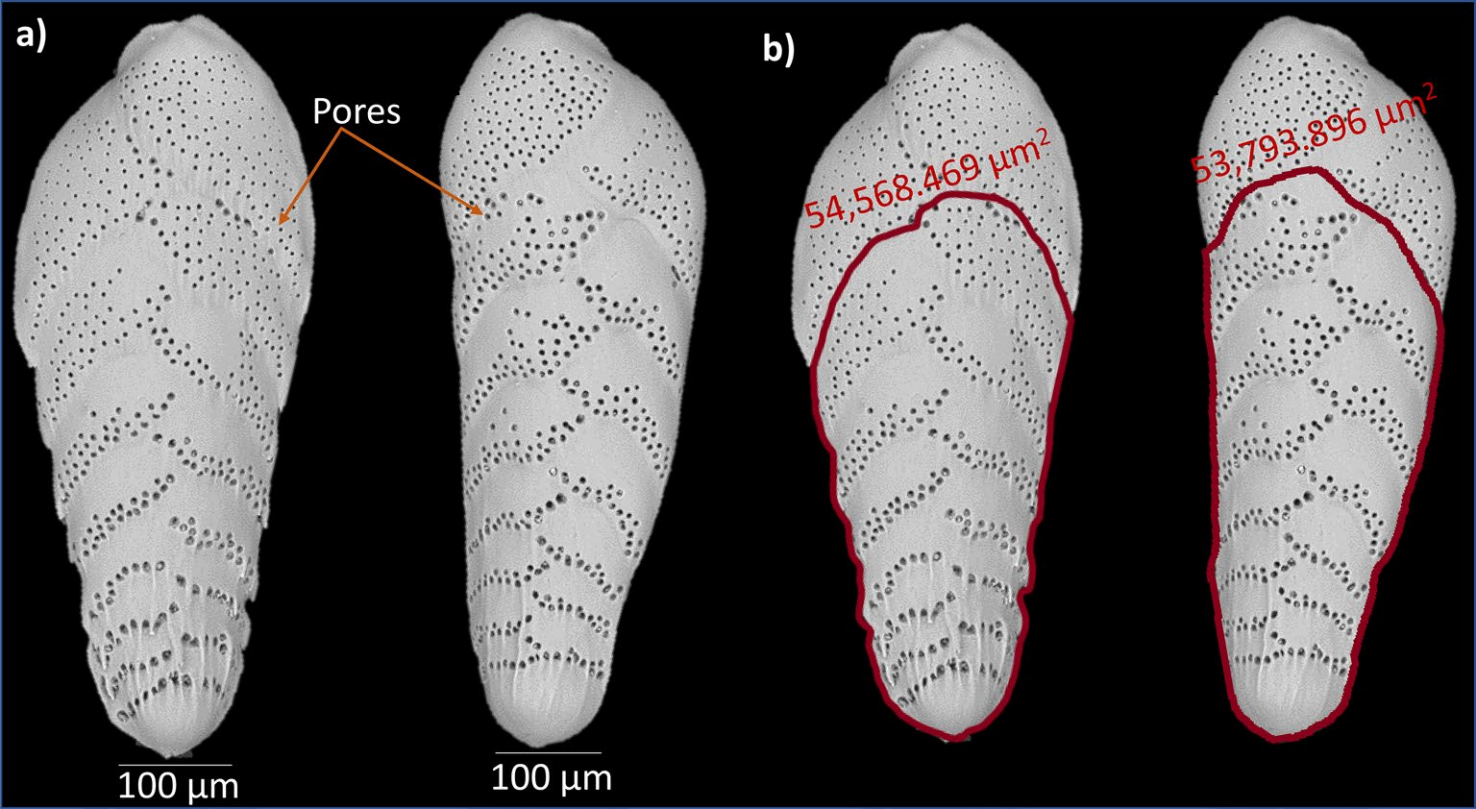
(**a**) Scanning electron microscopic images of *B. spissa* collected from Mexican Margin (MAZ-1E-04), (water depth: 1463 m) and (**b**) their total area relative to first (oldest) ~ ten chambers within 50,000–70,000 μm^2^ measured using ZEN lite software.

We determined pore parameters mean pore size, pore density, and porosity of the shallow infaunal species *Bolivina spissa* (see Fig. 1). Many bolivinids have an affinity for low-oxygen environments^42^. *Bolivina spissa* is well adapted to low oxygen conditions^32,43^, and has the ability to denitrify^41^, which makes it a promising species that might facilitate quantitative NO_3_^-^ reconstructions.

**Figure 2:**
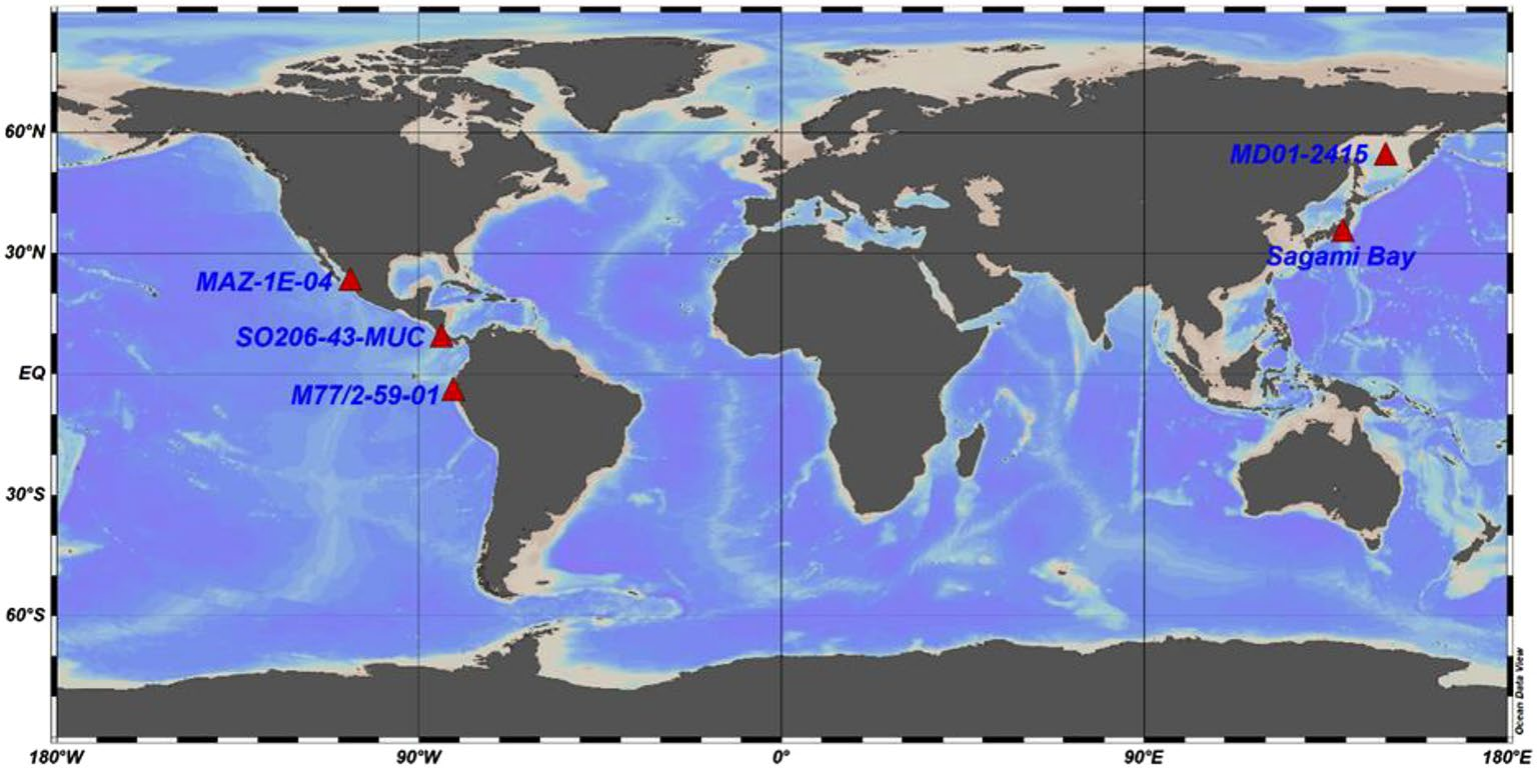
Map showing site locations studied: Gulf of Guayaquil (M77/2-59-01, depth: 997 m), Mexican Margin (MAZ-1E-04, depth: 1463 m), Sea of Okhotsk (MD01-2415, depth: 822 m), core-top samples from Costa Rica (Quepos Slide, SO206-43-MUC, depth: 568 m), and Sagami Bay (Japan, depth: 1410 m). The map was produced using Ocean Data View (Schlitzer, R., Ocean Data View, odv.awi.de, 2017).

We used foraminiferal specimens retrieved from five oxygen-depleted locations around the Pacific: the Gulf of Guayaquil (core M77/2-59-01), the Mexican Margin (core MAZ-1E-04), the Sea of Okhotsk (core MD01-2415), and “core-top” (i.e. surface-sediment) samples from Sagami Bay, and the continental margin of Costa Rica (Quepos Slide, core SO206-43-MUC) (Fig. 2). Here, we present a non-destructive, fast and statistically robust method for quantitatively describing the morphometrics in benthic foraminiferal tests. We applied an automated image recognition technique on scanning electron microscope (SEM) images using a deep learning algorithm to analyse the morphological features of *B. spissa*. Deep learning is a type of machine learning which is used to identify objects in images and allows to process data in a way according to user’s interest^44^.

We studied (1) the interdependence between the pore density, porosity and mean pore size of *B. spissa* to demonstrate whether total porosity is mainly influenced by the number or the size of pores and (2) whether porosity or pore density can be used as a robust proxy for bottom-water nitrate [NO_3_^–^]_BW_ reconstructions. Finally, we compare the pore density between *B. spissa*, *Bolivina subadvena, Bolivina subadvena accumeata,* and *Bolivina argentea,* and provide an extended nitrate vs. pore density calibration for *B. spissa* and *B. subadvena* from different locations around the Pacific.

## Results

### Comparison between manual and automated pore density analyses

Pore density measurements showed a 0–20% difference between manual and automated methods with an average individual difference at 4.2%. There was no significant difference in the mean pore density of all 31 specimens between the manual (0.0059 ± 0.0002 P µm^–2^; 1 SEM) and the automated (0.0059 ± 0.0002 P µm^–2^; 1 SEM) image analyses (T-test, p = 0.99). In three out of 31 cases the difference was 0% and the algorithm was counting exactly the same number of pores that have been recognized manually (Supplementary Table ST1). Only two specimens of *B. subadvena* showed a relatively high offset (10% and 20%). The original training of the algorithm is based on *B. spissa*. For future studies, which include a closer analysis of other species, we recommend an individual training for each species.

### Automated pore measurements with and without manual corrections

There was no significant difference for porosity (t = 0.31, p = 0.75) and pore density (t = 0.58, p = 0.56) obtained through automated image analysis with and without manual corrections, where artefacts of the automated image analyses were manually removed (Supplementary Tables ST2 and ST3).

### Interdependence between pore parameters of *B*. *spissa*.

The overall porosity values of all locations varied between 2.66% and 16.03% with a mean (± SD) of 8.52% (± 2.14%). The mean pore size varied between 5.98 µm^2^ and 47.62 µm^2^ with a mean (± SD) of 17.83 µm^2^ (± 3.83 µm^2^). The overall pore density varied between 0.002 P/µm^–2^ to 0.009 P/µm^–2^ with a mean (± SD) of 0.004 P/µm^–2^ (± 0.001 P/µm^–2^).

Specimens of *B. spissa* from the Gulf of Guayaquil, (M77/2-59-01) had the lowest porosity (7.14% ± 1.62%) and mean pore size (17.13 µm^2^ ± 4.37 µm^2^) of all analysed locations. The specimens from the Sea of Okhotsk, (MD01-2415) had the highest porosity (10.83% ± 1.66%) and mean pore size (20.67 µm^2^ ± 3.54 µm^2^). The mean pore density was not significantly different for the core-top samples (Costa Rica and Sagami Bay) and the down core samples from the Mexican Margin (MAZ-1E-04) and the Sea of Okhotsk. The pore density at the Gulf of Guayaquil (0.0043 P/µm^2^ ± 0.0008 P/µm^2^) was lower than at the other locations (Supplementary Table ST4).

**Figure 3:**
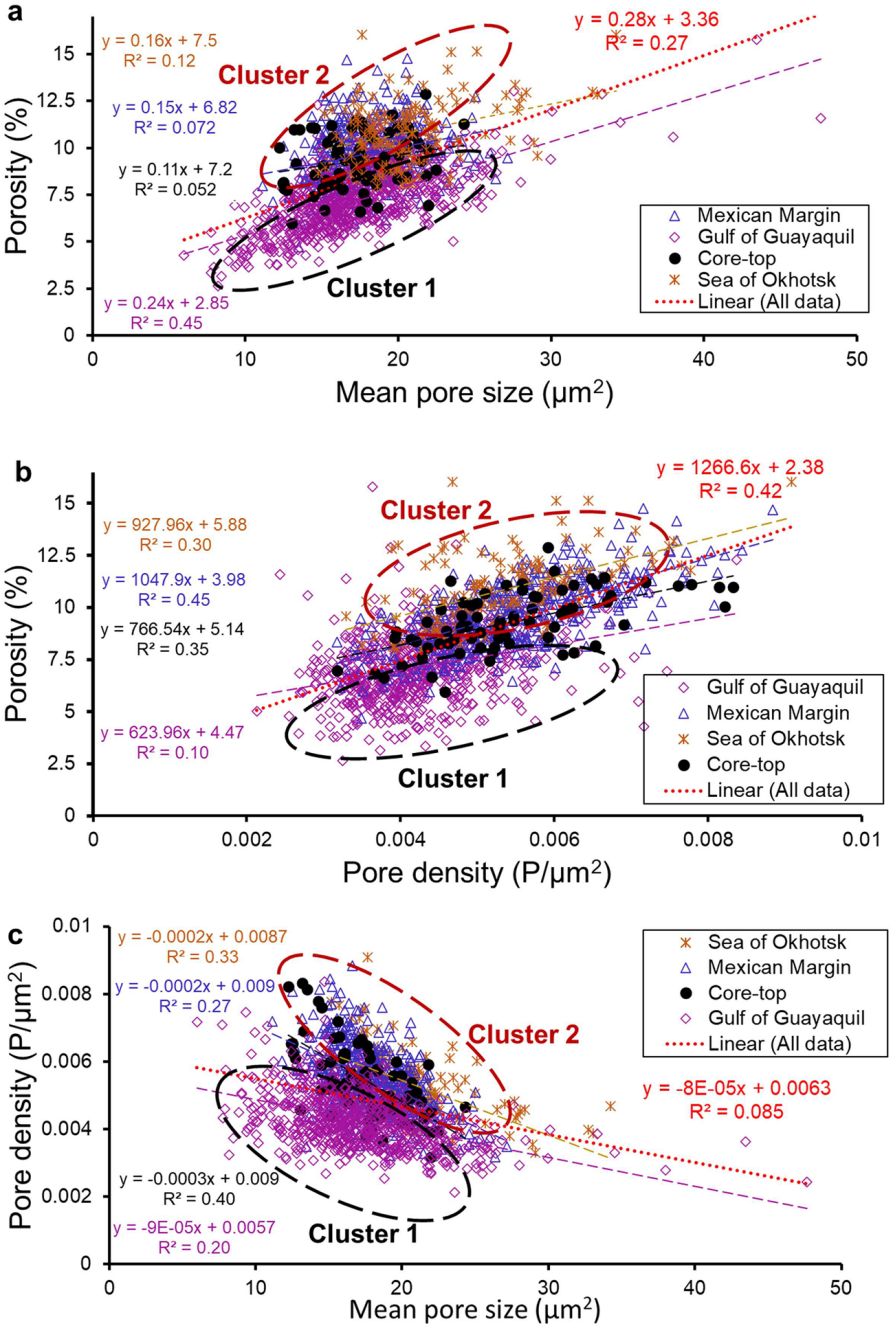
Relationship between (**a**) porosity vs mean pore size (**b**) porosity vs pore density (**c**) pore density vs mean pore size of *B. spissa* specimens from Gulf of Guayaquil (M77/2-59-01), Mexican Margin (MAZ-1E-04), Sea of Okhotsk (MD01-2415), and the core-top samples (Sagami Bay and Costa Rica). Total number of specimens utilized, n = 1344.

In general, there was a significant linear correlation between mean pore size and porosity (coefficient of determination, R^2^ = 0.27, p = 3.19E-93, Fig. 3a; Supplementary Table ST5) for all the analysed specimens. We observed strong regional differences in R^2^ among the studied sites. The R^2^ was highest for the specimens from the Gulf of Guayaquil (R^2^ = 0.45, p = 5.91E-89, Fig. 3a), and lowest for the specimens from core-top samples (R^2^ = 0.05, p = 0.047, Fig. 3a). We found a significant linear correlation between porosity and pore density (R^2^ = 0.42, p = 1.36E-15, Fig. 3b; Supplementary Table ST6) among all the sampling locations with the highest R^2^ of 0.45 at the Mexican Margin, while the specimens from the Gulf of Guayaquil showed the weakest correlation (R^2^ = 0.1, p = 3.21E-17, Fig. 3b) between porosity and pore density.

All analysed specimens showed a significant but weak negative linear correlation between pore density and mean pore size (R^2^ = 0.085, p = 1.34E-27, Fig. 3c; Supplementary Table ST7). We found a higher R^2^ for the core-top samples collected from Costa Rica and Sagami Bay (R^2^ = 0.4, p = 7.18E-10, Fig. 3c), and the weakest for the samples from the Gulf of Guayaquil (R^2^ = 0.20, p = 4.52E-35, Fig. 3c).

The combined data from all studied locations clearly fall apart into two distinguishable clusters for both porosity and pore density: “Cluster 1” (black dashed circle Fig. 3a), grouped most of the specimens belonging to the Gulf of Guayaquil (n = 669), and “Cluster 2” (red dashed circle, Fig. 3a), consisted of specimens belonging to the Mexican Margin (n = 445), the Sea of Okhotsk (144), and the core-top samples (n = 76). The porosity was significantly different between Cluster 1 and Cluster 2 (W = 50,716; p < 2.2e -16). This also accounts for the pore density (W = 79,726, p < 2.2e-16) and the mean pore size (W = 170,008; p = 4.49e-15). All data have been included in the Supplementary Table ST8.

### Inter-species comparison of pore parameters and pore density vs [NO_3_^–^]_BW_ calibration in the core-top samples

**Figure 4:**
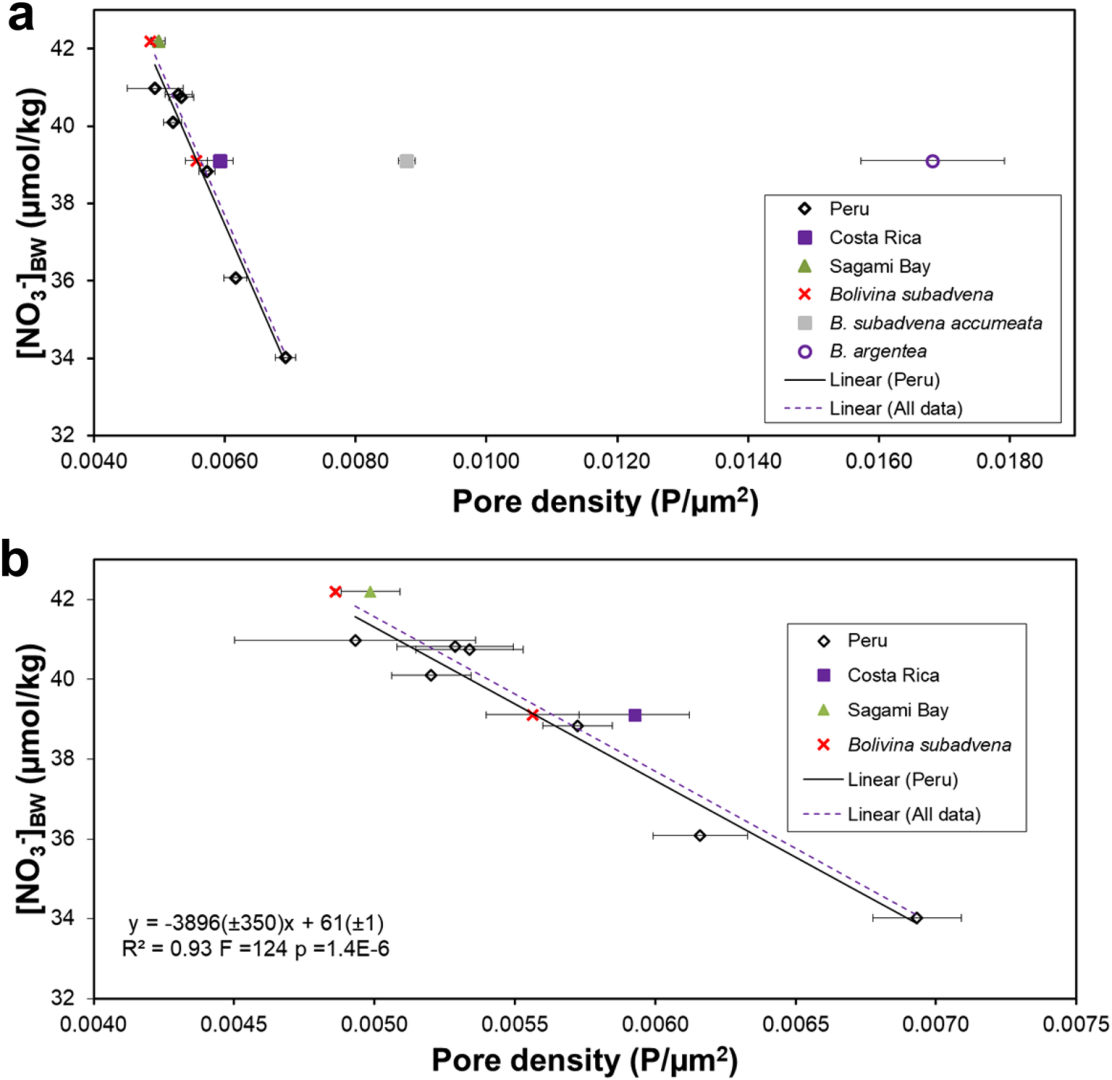
Correlation between the mean pore density of different closely related *Bolivina* species from core-top samples and [NO_3_^–^]_BW_. If no species name is indicated, the analysed species was *B. spissa*. The specimens of *B. subadvena*, *B. subadvena accumeata* and *B. argentea* are all from location SO206-43-MUC off Costa Rica, except the one specimen of *B. subadvena* at ~ 42 μmol/kg [NO_3_^–^]_BW_ that was collected at Sagami Bay (Japan). The linear fit (all data) has been applied to all available data for *B. spissa* and *B. subadvena*, except *B. subadvena accumeata* and *B. argentea*. (**a**) Pore density vs [NO_3_^–^]_BW_ plot including all analysed *Bolivina* species. (**b**) Pore density vs [NO_3_^–^]_BW_ plot only including *B. spissa* and *B. argentea*. The linear fit (Peru) alone was the published correlation from Glock et al.^32,45^ and only included *B. spissa* collected off Peru. Error bars are the standard error of the mean (1SEM).

While core-top specimens of *B. spissa* and *B. subadvena* from Costa Rica (Quepos Slide), and Sagami Bay (Japan) had a very similar pore density, pore densities of *B. subadvena accumeata* and *B. argentea* were around 50–300% higher (Fig. 4; Supplementary Table ST9). The new data for *B. spissa* and *B. subadvena* from Quepos Slide and Sagami Bay fit well into the pore density correlation with [NO_3_^–^]_BW_ that has been found for *B. spissa* from the Peruvian OMZ^32^ (Fig. 4). There was a highly significant linear correlation between the pore density of *B. spissa* and *B. subadvena* from Peru, Costa Rica, and Sagami Bay (R^2^ = 0.93, p < 0.0001, Fig. 4b). The data of *B. subadvena accumeata* and *B. argentea* were offset from this linear regression (Fig. 4a). The relationships between the pore density of *B. spissa* and *B. subadvena* from core-top samples (Costa Rica and Sagami Bay) and bottom-water oxygen (R^2^ = 0.43, p = 0.028; Supplementary Fig. SF1), temperature (R^2^ = 0.50, p = 0.015; Supplementary Fig. SF2), salinity (R^2^ = 0.41, p = 0.035; Supplementary Fig. SF3), and water depth (R^2^ = 0.48, p = 0.018; Supplementary Fig. SF4) has been analysed to test, if nitrate is the main factor that controls the pore density. These correlations are significant (R^2^ varies between 0.41 and 0.50; P varies between 0.015 and 0.035) but clearly weaker than the correlation of the pore density to nitrate (R^2^ = 0.93, p = 1.4E-6; Fig. 4b). The data for bottom-water oxygen, temperature, salinity and water depth from core-top samples have been included in Supplementary Table ST10.

Since pores were manually counted for the core-top pore density dataset off Peru from Glock et al.^32^, no data was available for the porosity of these specimens. A comparison of the porosity in tests of core-top samples of *B. spissa* from Costa Rica (9.5% ± 0.2%; 1SEM; N = 39) and Sagami Bay (9.1% ± 0.2%; 1SEM; N = 37) showed no significant difference between these two locations (p = 0.25). The Costa Rica [NO_3_^–^]_BW_ was lower and there was a significant difference in the pore density between these two locations (p = 8.7E-5, Fig. 4). This indicated that the pore density of *B. spissa* might be more sensitive to changes in the [NO_3_^–^]_BW_ than the porosity. In addition, while the pore density of *B. subadvena* fit very well into the pore density-[NO_3_^–^]_BW_ correlation of *B. spissa* (see Fig. 4), the porosity of *B. subadvena* was significantly higher than the porosity of *B. spissa* (10.9% ± 0.5% for *B. subadvena* vs. 9.5% ± 0.2% for *B. spissa* from Costa Rica; p = 0.0002).

## Discussion

### Evaluation of the automatic image recognition technique

Our study tested the application of a newly developed automated image recognition method for the detection of pore parameters of the benthic foraminiferal species *B. spissa*. This method can be used to accurately measure pore parameters such as the mean pore size, porosity, and pore density of *B. spissa*. This allows a high and efficient sample throughput (less than 1 min for one specimen) compared to manual analysis (5–6 min for one specimen) of pores. This automated deep learning approach produces results statistically identical to manual analyses. No significant improvement is found, if the results from the deep learning image analyses are manually corrected by removing artefacts from the images.

Both manual determination of pores using SEM images^32,46–48^ and automated measurements^49,50^, have advantages and disadvantages. For example, manual methods can be laborious and time-consuming. The fully automated method by Tetard et al.^50^ is rapid, allows quick generation of data, and the image acquisition and processing require no monitoring, however, it needs a very specific setup and is destructive, since the specimens are broken to shards. The semi-automatic method by Petersen et al.^49^ can produce reliable data in a short amount of time, minimizes artefacts related to the curvature of the tests, and gives information on pore area, perimeter, and circularity indexes but focuses only on a small part of the shell, which limits the amount of data per specimen.

By contrast, porosity measurements using deep-learning as applied in this study are non-destructive and automatically determines various pore parameters on the fully visible test surface. Moreover, the fully automated method is reproducible in comparison to manual methods where the analyses are performed by different operators. The application of a non-destructive method allows the use of the foraminifera for other analyses, thereby providing the possibility to use a single sample population for a multiproxy paleo reconstruction.

Although this automated method generates large datasets, proper attention should be given to the processing of curved specimens of *B. spissa*, because the curvature can create difficulties in counting the exact number of pores. Therefore, we suggest utilizing specimens with flat surfaces.

### Variation of pore patterns in *B. spissa* from different environments

All specimens of *B. spissa* that have been analysed showed a positive but weak correlation between the porosity and the mean pore size (R^2^ = 0.27, p < 0.05; Fig. 3a). Certain foraminifera species increase their porosity by increasing the size of their pores to facilitate electron acceptor uptake from the environment^49,51^. The strongest correlation between mean pore size and porosity at the Gulf of Guayaquil (M77/2-59-01) suggests that individuals at this location tend to increase the porosity by increasing their mean pore size rather by increasing its pore density. Similar observations were documented on *Ammonia* spp. that typically dwells in shallow marine environments such as tidal mudflats^25^. These species tend to increase their porosity by building fewer but larger pores, which has been suggested to ensure optimal shell stability^34,49^. The notable weaker correlation between porosity and mean pore size, for the other analysed sites (R^2^ between 0.05 and 0.12, Fig. 3a) implies that most of the analysed *B. spissa* do not control their porosity by modifying the size of the pores. This weak correlation between porosity and the mean pore size in *B. spissa* is an indicator that the size of the pores is only a secondary control on overall porosity of *B. spissa* at most of the studied locations.

The strongest significant linear correlation between porosity and pore density has been found at the Mexican Margin (MAZ-1E-04) (Fig. 3b), which suggests that *B. spissa* adjusts its porosity by adapting the number of pores and not the pore-size. Specimens from the Gulf of Guayaquil are exceptional as they show only a weak correlation between porosity and pore density (R^2^ = 0.1, Fig. 3b). Nevertheless, the negative correlation between pore density and mean pore size among the studied sites (Fig. 3c) are in good agreement with previous studies on *Ammonia* spp.^34,49^. Mechanical constraints like shell stability could be a controlling factor leading to the inverse relationship between pore density and mean pore size^34^. Our new data shows that, except in the Gulf of Guayaquil, *B. spissa* mainly controls its porosity by the number of pores.

The different trends at different locations indicate that long-term environmental conditions or genetic factors likely play a pivotal role in contributing to the morphological differences in benthic foraminifera since the sediment cores cover periods of ~ 20 kyrs. Especially at the Gulf of Guayaquil, the pore parameters showed significant differences to the other studied locations. We speculate that these differences could be related either to the mechanism of electron acceptor uptake or to genetic factors. Benthic foraminifera can actively migrate within the sediment to their preferred microhabitat^52–54^ which exposes them to an oxygen/nitrate concentration gradient. The habitat preference of *B. spissa* in oxygen-deficient zones necessitates the use of alternate electron acceptors like nitrate for respiration^41^. In nitrate-depleted habitats, *B. spissa* optimizes its nitrate accumulation by building more pores to efficiently take up nitrate resulting in higher pore density^32^. Previous observations found that the cell size of many denitrifying foraminifers is limited by nitrate availability instead of oxygen^41^. Several denitrifying foraminiferal species, including *B. spissa*, have been shown to encode a NO_3_^–^ transporter in their genome and transcriptome^55,56^. This means by using these NO_3_^–^ transporters they can actively pump NO_3_^–^ into their cells, since NO_3_^–^ is a charged ion. This NO_3_^–^ can be stored as intracellular nitrate (ICN) which can be utilized as a source of energy for metabolic activities^21,40,57–59^ via complete denitrification during oxygen-depleted conditions.

### Biogeochemical controls on the pore patterns in the Gulf of Guayaquil

The site from where core M77/2-59-01 was retrieved (3.95° S, 81.23° W) is outside core oxygen minimum zone off Peru. The modern oxygen concentration recorded closest to this site is 55 µmol/kg, which is higher than at the other studied locations (38–47 µmol/kg)^60^. When oxygen concentration increases above a certain threshold, there will be less overall denitrification^61,62^ resulting in higher nitrate availability. We speculate that if there is more nitrate in the Gulf of Guayaquil relative to the other studied locations in the modern ocean, this was likely also the case in the past. This is supported by a sedimentary nitrogen isotope record on the same core M77/2-59-01 by Mollier-Vogel et al.^63^ and Mollier-Vogel et al.^64^, which indicated that pelagic denitrification was low at this location over the entire last deglaciation. The regional differences in the patterns at Gulf of Guayaquil could be an adaptation to the continuously higher nitrate availability at this site.

### Genetic controls on the pore patterns in the Gulf of Guayaquil

The *B. spissa* specimens from the Gulf of Guayaquil are, except for their pore characteristics, morphologically similar to the *B. spissa* from the other locations but could be a different phylogenetic strain. Observations of *Ammonia* specimens by Hayward et al.^46^ suggested that genetically different species can also be morphologically distinguished. Later studies found genetically well-separated species of the *Ammonia* genus, which have earlier been considered as eco-phenotypes of *Ammonia*, can now be morphologically distinguished by their pore patterns and other subtle morphological features^25^. Similarly, it is possible to have the existence of genetic variation and cryptic species within a *B. spissa* morpho-group due to the wide geographical distances, and variability in ecological conditions that separated oxygen-depleted regions in the Pacific. Nevertheless, the phylotypes of *B. spissa* without a combined morphometric molecular analysis would be very difficult to discriminate as a separate species.

### An extended modern pore density vs. nitrate calibration

Since there are studies that use either the pore density or porosity to reconstruct past environmental conditions^32,33,45,65,66^ we intended to address whether pore density or porosity is a better proxy for quantitative nitrate reconstructions. Although pore density in *B. spissa* shows a significant correlation to nitrate (Fig. 4b), the correlation between porosity and nitrate availability has not been systematically tested, yet. In addition, an extension of the local nitrate vs. pore density calibration for the Peruvian OMZ^32^ to other regions and foraminiferal species would increase the applicability of this proxy.

**Figure 5:**
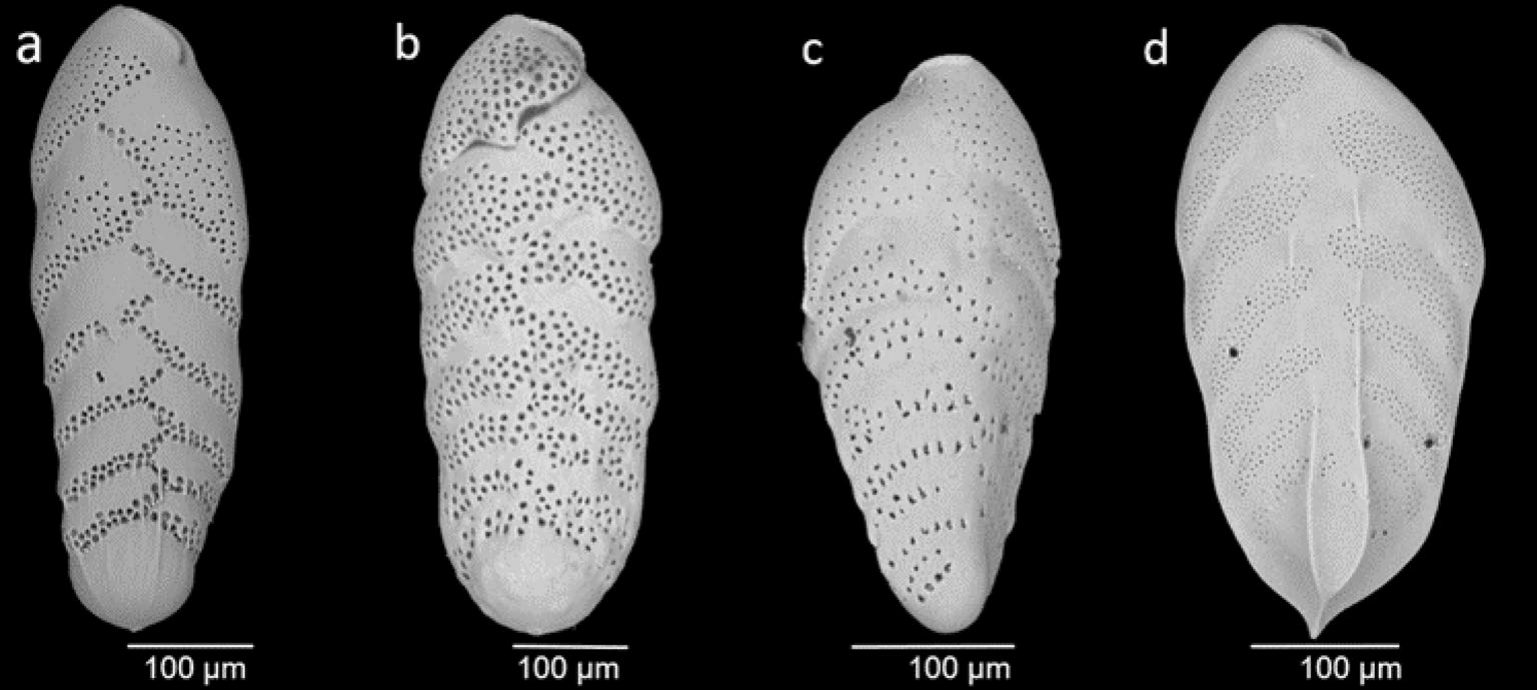
Scanning electron microscopic images of bolivinids (**a**) *B. spissa*, (**b**) *B. subadvena* (**c**) *B. subadvena accumeata*, and (**d**) *B. argentea*.

Figure 4 shows the relationship between pore density in other bolivinids and [NO_3_^–^]_BW_ from core-top samples at different locations of the Pacific. The linear correlation between the pore density of *B. spissa* and *B. subadvena* and [NO_3_^–^]_BW_ is highly significant and much stronger than the correlation to oxygen, temperature, salinity or water depth (Supplementary Figs. SF1 to SF4), making their pore density a promising proxy for present and past [NO_3_^–^]_BW_. This also suggests a close phylogenetic relationship with similar metabolic adaptations of both species. Indeed, *B. spissa* was originally classified as a variant of *B. subadvena* with the name *B. subadvena* var. *spissa*^67^ and 7 out of 7 *Bolivina* species that have been tested for denitrification were able to denitrify and 11 out of 12 analysed species intracellularly stored nitrate [Ref.^68^ and references therein]. Although *B. subadvena accumeata* is still considered a subspecies of *B. subadvena*, the pore characteristics are distinct from either *B. spissa* or *B. subadvena*. The pore density of *B. argentea* is elevated compared to the other species as it tends to build numerous but very small pores (see Fig. 5).

Therefore, the pore density of *B. spissa* and *B. subadvena* both can be used to reconstruct past [NO_3_^–^]_BW_ conditions according to the following equation (Eq. 1), However, *B. subadvena accumeata* and *B. argentea* should be avoided, when the calibration shown in Eq. (1) is used. Future studies will show, if the latter two species also show species-specific relationships that might be used for paleoceanographic reconstructions.

1 $$\left[ {{\text{NO}}_{{3^{ - }}}} \right]_{{{\text{BW}}}} = \, - {3896 }\left( { \pm {35}0} \right){\text{ PD }} + { 61}\left( { \pm {1}} \right),$$

where PD is the pore density.

While the pore characteristics of denitrifying foraminifera are promising paleoproxies for past [NO_3_^–^]_BW_^32,45^, pore characteristics of the epifaunal species *Cibicidoides* and *Planulina* spp. that likely rely on O_2_ respiration seem to be good indicators for past bottom-water oxygen concentration [O_2_]_BW_^33,65^. Intriguingly, while the new data on *B. subadvena*, and *B. spissa* indicate that pore density is more sensitive to ambient [NO_3_^–^] variations than the total porosity, it appears that the opposite is the case for epifaunal species. In *Cibicidoides* and *Planulina* spp. porosity is more sensitive to ambient [O_2_] fluctuations than the pore density^33,65^.

Data from only two sites for the correlation between total porosity of bolivinids and [NO_3_^–^]_BW_ are available. Future studies should address this issue and include both the pore density and total porosity. The fact that porosity of *B. spissa* from the Sagami Bay and Costa Rica core-tops are similar, but the pore density at Costa Rica is significantly higher indicates that the Sagami Bay specimens build larger pores than the specimens from Costa Rica.

The different pore characteristics of denitrifying bolivinids and the aerobic epifaunal species might be related to the mechanism of electron acceptor uptake. The uptake of O_2_ is limited by passive diffusion, since O_2_ is not charged and foraminifera have no respiratory organs that can actively take up O_2_. Thus, aerobic foraminifera can only increase the O_2_ uptake through the pores by increasing the area of pores on their test (i.e. total porosity), which can be done by either creating more pores (increase in pore density) or larger pores (increase in mean pore size). Some foraminifera species ensure better shell stability by increasing their porosity through building less but larger pores^34^. Thus, the increase of total porosity of epifaunal *Cibicidoides* and *Planulina* spp. might also be restricted by shell stability. They tend to build larger pores to increase their porosity, which might explain the weaker correlation between pore density and ambient [O_2_] compared to total porosity^33,45^. Denitrifying bolivinids can actively pump NO_3_^–^ into their cells, since NO_3_^–^ is a charged ion and they genetically encode nitrate transporters^55,56^. Thus, we hypothesize that the denitrifying bolivinids do not rely on the increase of total porosity but rather on the number of pores to enhance electron acceptor uptake. For the moment, the empiric correlation between the pore density of *B. spissa* and *B. subadvena* appears to be solid, since a deglacial pore density record of *B. spissa* from the Peruvian margin reconstructed similar [NO_3_^–^]_BW_ as other proxies and various modeling studies^45^.

## Conclusions

The application of automated image analysis through deep-learning provided a robust method for determining the pore patterns in the shallow infaunal benthic foraminiferal species *B. spissa*. The differences in pore patterns of *B. spissa* found between different studied locations suggest caution in the interpretation of the results. Nevertheless, our new data shows that, except for the Gulf of Guayaquil, *B. spissa* mainly controls its porosity by the number of pores. This gives additional validation that the pore density of *B. spissa* is a robust and reliable paleo-proxy for nitrate concentrations in bottom-waters. Quantitative reconstructions of past bottom-water nitrate concentrations could help us to predict the environmental and ecological impacts of future climate scenarios. Moreover, understanding the factors controlling porosity in bolivinids provides insight into benthic denitrification, which is indispensable for future biogeochemical studies. Future studies concerning foraminiferal porosity should consider both mean pore size and pore density, and a combined morphometric molecular approach for the complete description of foraminiferal pore patterns. As the presence of cryptic species within a morphogroup might complicate paleoceanographic interpretation of pore density or porosity in benthic foraminifera, the phylogenetic analyses of *Bolivina* species is highly relevant for better proxy validations.

## Methods

### Sampling of sediment cores

The piston core M77/2-059-1 (03° 57.01′ S, 81° 19.23′ W, recovery 13.59 m) was retrieved from the Gulf of Guayaquil at 997 m water depth during RV Meteor cruise M77/2 in 2008. The chronostratigraphy is based on accelerator mass spectrometry radiocarbon dating (AMS^14^C) of planktonic foraminifers, supported by benthic stable oxygen isotope (δ^18^O) stratigraphy from *Uvigerina peregrina*^69,70^. The CALYPSO giant piston core MD01-2415 (53° 57.09′ N, 149° 57.52′ E, recovery 46.23 m) was recovered from the northern slope of the Sea of Okhotsk at 822 m water depth during WEPAMA cruise MD122 of the R/V Marion Dufresne^71,72^. The chronostratigraphic framework of core MD01-2415 is based on a combination of stable oxygen-isotope stratigraphy, AMS^14^C dating, and orbital tuning^72^. The piston core MAZ-1E-04, Mexican Margin was collected on board the RV El Puma at a water depth of 1468 m. The core, SO206-43-MUC was retrieved in 2009 from a sea mound slope (Quepos Slide) off Costa Rica during RS Sonne cruise SO206 using a multicorer. Supernatant water of the multicorer SO206-43-MUC was carefully removed. Then, the core was gently pushed out of the multicorer tube. For the foraminiferal analyses, the core was cut into 10 mm thick slices (upto 20 cm depth) and samples were transferred to Whirl-Pack™ plastic bags and stored at a temperature of 4 °C.

The sediment samples from central part of Sagami Bay were collected by a push core (inner diameter: 8.2 cm, tube length: 32.0 cm) using the manipulator of human occupied vehicle *Shinkai6500* in 2021 (Table 1 shows the details of all sampling locations). The surface 2 cm of the sediment was subsampled by extruding from the push core tube and then kept frozen prior to an isolation of foraminifera. Bottom-water temperature, salinity, and dissolved oxygen concentrations were 2.3 °C, 34.5, and 56.4 µM, respectively, which were measured with the CTDO sensor (Seabird SBE19).

**Table 1:** Site location information and distribution of specimens (*B. spissa*) from different sampling locations used in the study.

Locations	Latitude	Longitude	Water depth (m)	No of *B. spissa* specimens
Gulf of Guayaquil, (M77/2-59-01)	3.95° S	81.32° W	997	669
Mexican Margin, (MAZ-1E-04)	22.9° N	106.91° W	1468	455
Sea of Okhotsk, (MD01-2415)	53.95° N	149.96° E	822	144
Sagami Bay push core	35.09° N	135.38° E	1410	37
Costa Rica, (SO206-43-MUC)	8.87° N	84.23° W	568	39

### Sample processing

All sediment samples from the Gulf of Guayaquil (M77/2-59-01), Mexican Margin (MAZ-1E-04), Sea of Okhotsk (MD01-2415), Costa Rica (SO206-43-MUC), and the Sagami Bay were washed and wet-sieved through a 63 µm mesh sieve. The residues were dried in an oven at temperatures between 38 and 50 °C. Afterwards the samples were fractioned into the grain-size fractions of 63–125, 125–250, 250–315, 315–355, 355–400, and > 400 μm. Specimens of *Bolivina spissa*, *Bolivina subadvena*, *Bolivina subadvena accumeata* and *Bolivina argentea* were picked from the 125–250 μm fraction. Only megalospheric specimens of *B. spissa*, were used for the pore analysis.

### Bottom-water nitrate analyses at core-top locations

Supernatant water was sampled for the analysis of bottom-water NO_3_^–^ concentrations in a core replicate from the multicore deployment at Costa Rica, (SO206-43-MUC). For the bottom water sample, a total of 2 ml was passed through a cadmium (Cd) catalyst to reduce NO_3_^–^ to NO_2_^–^ (nitrite), which was then analysed on-board using photometry. The resulting concentration is a mixture of NO_3_^–^, and NO_2_^–^. Since NO_2_^–^ is a transient intermediate species in the benthic nitrogen cycle and is generally present at lower concentrations than NO_3_^–^, the NO_2_^–^ concentration determined is assumed to approximately represent the concentration of NO_3_^–^.

For Sagami Bay nitrate analyses, ~ 20 mL of overlying water was gently collected using a tube. The overlying water was filtered through a 0.45 µm membrane filter and then stored at − 25 °C before nutrient analyses back in land-based laboratory. Nutrient concentrations were measured with a continuous-flow analyzer (BL-Tech QUAATRO 2-HR system, Japan)^73^. The data for the Peruvian OMZ cores has been taken from^32^.

### Bottom-water salinity, temperature and oxygen at core-top locations

Bottom-water conditions at the locations that have been used for the core-top calibrations are shown in Table 2. Salinity, oxygen and temperature for the Costa Rica core have been taken from the World Ocean Atlas location 24,671(B), 84.5° W, 8.5° N and 550 m depth^60^. At the Sagami Bay location bottom-water temperature, salinity, and dissolved oxygen concentrations were measured with the CTDO sensor (Seabird SBE19). Data for bottom-water oxygen and temperature at the locations from the Peruvian OMZ were taken from^32^. Salinity data for the Peruvian OMZ was taken from^74^, using the CTD-data at M77/1-501/CTD-RO-23.

**Table 2:** Bottom-water conditions at the sampling locations that have been used for the core-top calibration.

Sampling locations	Nitrate (µM)	Water depth (m)	Salinity	Oxygen (µmol/kg)	Temperature (°C)
Costa Rica (SO206-43-MUC)	39.1	568	34.69	9.53	7.47
Sagami Bay push core (Japan)	42.2	1410	34.50	56.40	2.30
*M77/1-455/MUC-21 (OMZ, Peru)*	34.0	465	34.64	2.42	8.12
*M77/1-565/MUC-60 (OMZ, Peru)*	40.1	640	34.56	8.17	6.70
*M77/1-445/MUC-15 (OMZ, Peru)*	40.8	928	34.56	36.77	4.76
*M77/1-487/MUC-39 (OMZ, Peru)*	38.8	579	34.55	3.70	7.21
*M77/1-459/MUC-25 (OMZ, Peru)*	41.0	698	34.57	12.55	6.68
*M77/1-604/MUC-74 (OMZ, Peru)*	40.8	878	34.53	34.23	5.72
*M77/1-516/MUC-40 (OMZ, Peru)*	36.1	513	34.60	2.40	8.05

Sampling locations in italic letters have been taken from^32^.

### Image acquisition

A total number of 23 sample depths from the Mexican Margin (MAZ-1E-04), 37 sample depths from the Gulf of Guayaquil (M77/2-59-01), 12 sample depths from the Sea of Okhotsk (MD01-2415), and 2 core-top samples from Sagami Bay (Japan) and Costa Rica, (SO206-43-MUC) were utilized. All specimens of *B. spissa* were mounted onto carbon pads and photographed using Scanning Electron Microscope (version: Hitachi Tabletop SEM TM4000 series). All images were captured at a magnification of 150x. Due to the more or less flat surface of *B. spissa*, pore openings were generally well-defined, and clearly distinguishable from the SEM images. The total area on the tests of the specimens were determined using the Zeiss ZEN lite software (version: ZEN 3.4 blue edition; https://www.zeiss.com/microscopy/de/produkte/software/zeiss-zen-lite.html).

### Size normalization

To reduce ontogenetic effects, the total area equivalent to the first (oldest) ~ ten chambers (covering 50,000–70,000 µm^2^) were measured for the quantification of pore parameters^32^. The pore density increases with each newly built chamber (Fig. 5**),** related to a decrease in the surface/volume ratio with the size of the specimens. If the more recent 1–2 chambers would be analysed, only specimens within the same ontogenetic stage could be used. i.e. the size of the specimen and the number of chambers should be the same in all the chosen specimens. It is practically impossible to use only specimens having exactly the same number of chambers. By sticking to the oldest chambers of the foraminifer the ontogenetic effects are minimized by size normalization. Considering the short life span of foraminifera, the data from earlier ontogenetic stages still provide a proper representation of the present situation.

Moreover, the larger area provides a statistically robust, and larger dataset for each analysed specimen^65^.

### Automated image analysis

A total number of 1344 fossil specimens of *B. spissa* sampled from five different sampling locations were analysed. Porosity measurements were made on 6–20 well-preserved specimens of *B. spissa* in each of the studied locations. The pore density, mean pore size, and porosity were determined with an automated image analyzing software Amira (version: Amira^TM^ 3D pro) using a previously trained deep-learning algorithm. The deep learning algorithm that has been used for this study is included in the Amira software package. We used a convolutional neural network model (UNet) backboned with a resnet18 model for the deep learning training. The deep learning algorithm was trained with manually segmented pores on 52 images of *B. spissa*. In total 17,649 pores have been segmented manually for the deep learning training.

Only those specimens that had a total area equivalent to at least 50,000 µm^2^ were used for the automated analysis. The main steps for porosity measurements in Amira were:

- Import of multiple SEM images.
- The deep learning algorithm to recognize the pores was applied on imported images.
- Only the oldest chambers that fit within the total area of 50,000 to 70,000 µm^2^ were taken into acccount (Fig. 1b). All chambers beyond this threshold were manually removed, using the segmentation tools in the Amira software.
- A table with all measured pore characteristics can be exported by the software at the end of each set of analysis.

### Comparison of manual vs. automatic pore density determination

To assess the reliability of the deep learning algorithm pore density was determined manually for 31 specimens belonging to the species *B. spissa* (27 specimens) *B. subadvena* (3 specimens) and *B. subadvena accumeata* (1 specimen). For four additional specimens of *B. argentea* pore density was determined manually, since the pores in this species are very small and not recognized by the deep learning algorithm that was trained with images of *B. spissa*. The detailed procedure for manual pore density determinations are published in Glock et al.^32^.

### Automated pore measurements with and without manual corrections

To explore whether manual corrections (i.e. corrections done on the specimens that were automatically pore analysed) made a significant difference on automated data, a total number of 858 specimens were randomly selected and analysed both with and without manual correction. To apply manual corrections, we removed all artefacts (i.e. unwanted particles on the surface of *B. spissa*) on each specimen during the automated image analysis and obtained porosity data. For the automated image analysis without manual corrections, we applied the method of analyzing each specimen without manually removing the artefacts. Statistical analysis was carried out to decide if the porosity data obtained through either of these methods were significantly different or not. The data have been included as supplementary information in Supplementary Tables ST2 and ST3.

The preliminary statistical analysis was carried out in Excel and verified using R. To test the normality of the samples, we used Shapiro–Wilk normality test whenever necessary. To determine the correlation between pore parameters, a linear ordinary least-square regression was used. For normal distributions, we used the parametric Student’s *t* test (t), and for non-normal distributions we used the non-parametric Wilcox test (W). All the data generated or analysed during this study have been included in the supplementary information files.

### Supplementary Information

Supplementary Table S1.

Supplementary Table S8.

Supplementary Table S9.

Supplementary Table S10.

Supplementary Information.

## Data Availability

All data generated or analysed during this study are included in the tables of this published article (and its Supplementary Information files).
