# Supplementary material for: A deep-learning automated image recognition method for measuring pore patterns in closely related bolivinids and calibration for quantitative nitrate paleo-reconstructions
Source: Sci Rep. 2023 Nov 10;13:19628. doi: 10.1038/s41598-023-46605-y (PMC10638366; doi:10.1038/s41598-023-46605-y)
Supplement: Supplementary file 5 — Supplementary Information. [file 41598_2023_46605_MOESM5_ESM.pdf]

A deep-learning automated image recognition method for measuring pore patterns in closely related bolivinids and calibration for quantitative nitrate paleo-reconstructions

Anjaly Govindankutty Menon[1*], Catherine V. Davis[2], Dirk Nürnberg[3], Hidetaka Nomaki[4], Iines Salonen[4], Gerhard Schmiedl[1,5], Nicolaas Glock[1]

[1]*Department of Earth System Sciences, Institute for Geology, Universität Hamburg, Bundesstrasse 55, D-20146 Hamburg, Germany*

[2]*Department of Marine, Earth, and Atmospheric Sciences, North Carolina State University, 2800 Faucette Dr, Raleigh, NC, 27695, United States*

[3]*GEOMAR Helmholtz Centre for Ocean Research Kiel, Wischhofstr. 1-3, Geb. 8c, Raum 106, D-24148 Kiel, Germany*

[4]*SUGAR, X-star, Japan Agency for Marine-Earth Science and Technology (JAMSTEC), 2-15 Natsushima-cho, Yokosuka 237-0061, Japan*

[5]*Center for Earth System Research and Sustainability, Institute for Geology, Universität Hamburg, Bundesstrasse 55, D-20146 Hamburg, Germany*

**Corresponding author: anjaly.govindankutty.menon@uni-hamburg.de*

Supplementary Figures

Supplementary Figure SF1. Relationship between the mean pore density of different closely related *Bolivina spissa* and *Bolivina subadvena* from core-top samples (Costa Rica and Sagami Bay) and oxygen concentration (R^2 = 0.43, p = 0.028). The error bars are the standard error of the mean (1SEM).

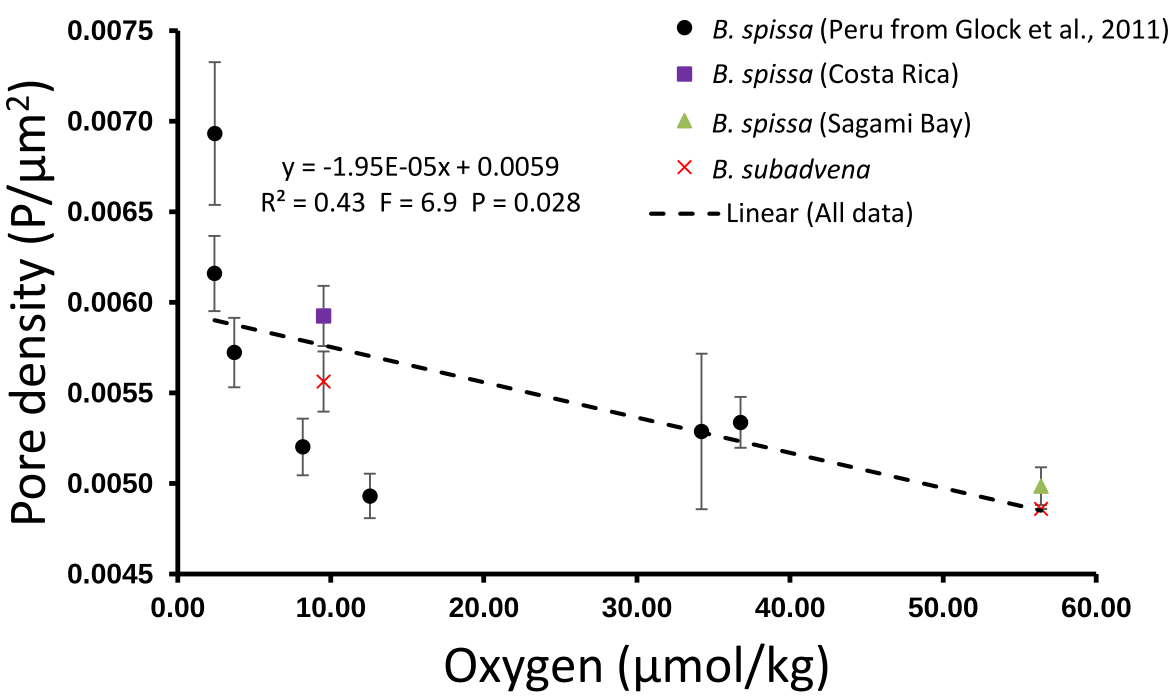

Supplementary Figure SF2. Relationship between the mean pore density of different closely related *Bolivina spissa* and *Bolivina subadvena* from core-top samples (Costa Rica and Sagami Bay) and temperature (R^2 =0.50, p = 0.015). The error bars are the standard error of the mean (1SEM).

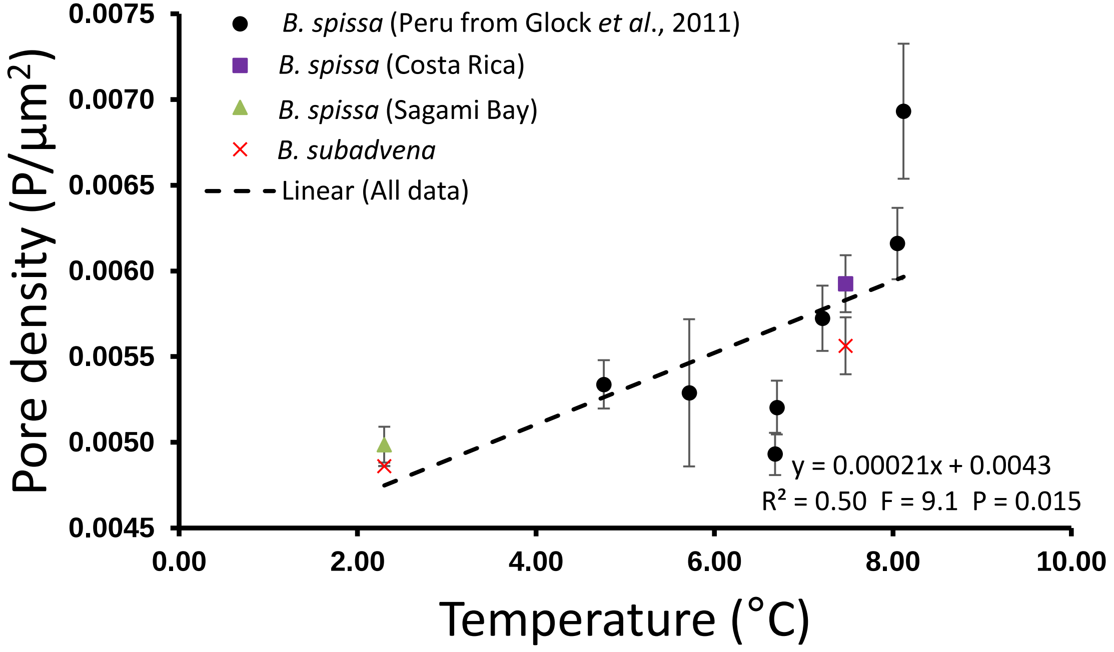

Supplementary Figure SF3. Relationship between the mean pore density of different closely related *Bolivina spissa* and *Bolivina subadvena* from core-top samples (Costa Rica and Sagami Bay) and salinity (R^2 =0.41, p = 0.035). The error bars are the standard error of the mean (1SEM).

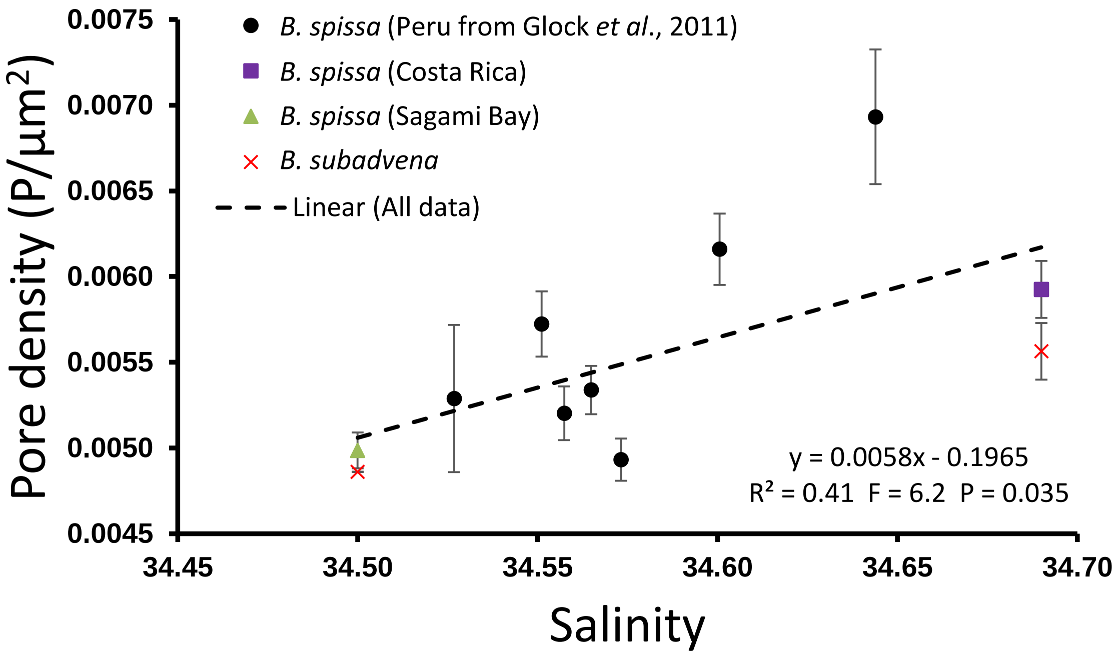

Supplementary Figure SF4. Relationship between the mean pore density of different closely related *Bolivina spissa* and *Bolivina subadvena* from core-top samples (Costa Rica and Sagami Bay) and water depth. The error bars are the standard error of the mean (1SEM).

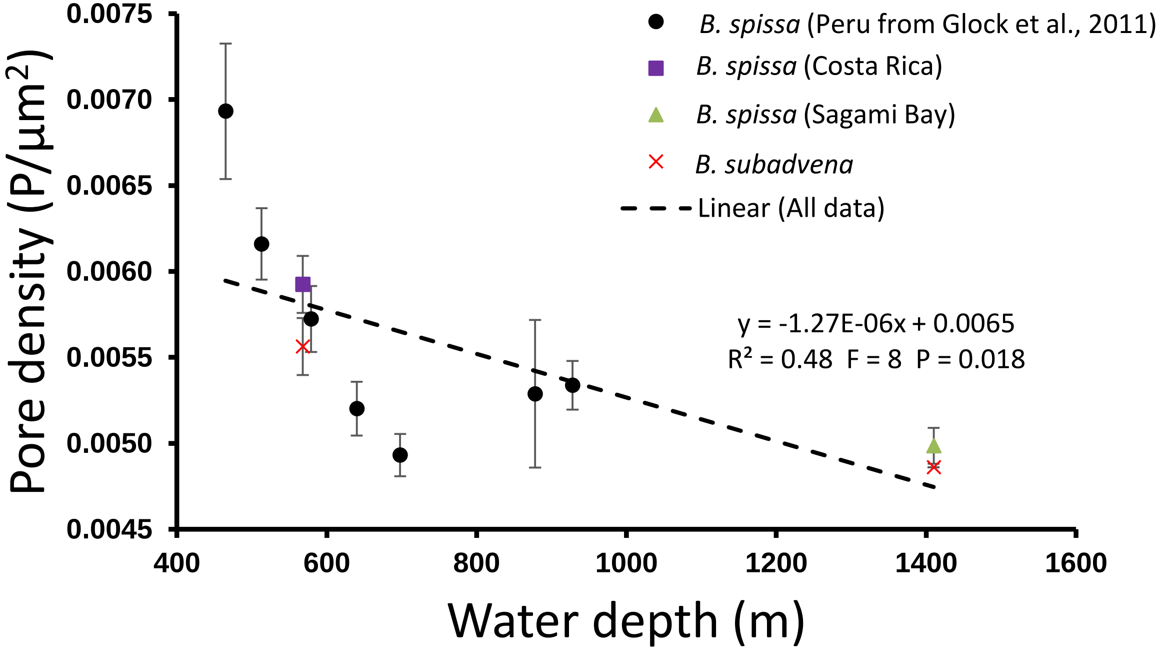

Supplementary Tables

Supplementary Table ST1. Details of manual and automatic (deep-learning) pore density calculations of a total of 31 specimens belonging to species *B. spissa* (27 specimens) *B. subadvena* (3 specimens) and *B. subadvena* accumeata (1 specimen) from core-top samples (Costa Rica and Sagami Bay).

Supplementary Table ST2. Results of statistical analysis on pore measurements with manual corrections (Total number of pores =8,194; number of specimens = 858)

Supplementary Table ST2				
Depth (cm)	Location	Pore density (P/µm^2)	Mean pore area (µm^2)	Porosity (%)
8	Gulf of Guayaquil	0.0042	19.82	8.31
53	Gulf of Guayaquil	0.0042	19.16	7.99
588	Gulf of Guayaquil	0.0043	15.42	6.60
18.5	Mexican Margin	0.0057	18.41	10.43
43.5	Mexican Margin	0.0056	19.63	10.92

Supplementary Table ST3. Results of statistical analysis on pore measurements without manual corrections (Total number of pores = 7,788; number of specimens = 858)

**Supplementary Table ST3**				
Depth (cm)	Location	Pore density ($P/\mu m^2$)	Mean pore area (μm^2)	Porosity (%)
8	Gulf of Guayaquil	0.0041	20.09	8.2
53	Gulf of Guayaquil	0.0041	19.58	7.97
588	Gulf of Guayaquil	0.0042	15.31	6.37
18.5	Mexican Margin	0.0055	18.94	10.3
43.5	Mexican Margin	0.0053	20.39	10.78

Supplementary Table ST4. Statistical results of mean + standard deviation (SD) of all pore parameters of *B. spissa* all locations used.

**Supplementary Table ST4**				
Pore parameter	Mean ± SD Gulf of Guayaquil	Mean ± SD Mexican Margin	Mean ± SD Sea of Okhotsk	Mean ± SD Core-top samples
Pore density	0.0043 ± 0.0008	0.0054 ± 0.0009	0.0053 ± 0.0009	0.0054 ± 0.001
Porosity	7.14 ± 1.62	9.69 ± 1.56	10.83 ± 1.66	9.33 ± 1.41
Mean Pore area	17.13 ± 4.37	18.03 ± 2.63	20.67 ± 3.54	17.41 ± 2.73

Supplementary Table ST5. Results of linear regression tests performed between porosity and mean pore size measurements of *B. spissa* from different studied locations.

Supplementary Table ST5			
Location	R square	p value	Significance
Gulf of Guayaquil	0.45	5.91E-89	P < 0.05
Mexican Margin	0.072	6.86E-09	P < 0.05
Sea of Okhotsk	0.12	2.56E-05	P < 0.05
Core-top samples	0.052	0.047	P < 0.05
All data	0.27	3.19E-93	P < 0.05

Supplementary Table ST6. Results of linear regression tests performed between porosity and pore density measurements of *B. spissa* from different of studied locations.

Supplementary Table ST6			
Location	R square	P value	Significance
Gulf of Guayaquil	0.1	3.2141E-17	P < 0.05
Mexican Margin	0.45	3.45E-60	P < 0.05
Sea of Okhotsk	0.3	7.46E-13	P < 0.05
Core-top samples	0.35	2.34E-08	P < 0.05
All data	0.42	1.36E-15	P < 0.05

Supplementary Table ST7. Results of linear regression tests performed between pore density and mean pore size measurements of *B. spissa* from different studied locations.

Supplementary Table ST7			
Location	R square	p value	Significance
Gulf of Guayaquil	0.2	4.52E-35	P < 0.05
Mexican Margin	0.27	4.2E-33	P < 0.05
Sea of Okhotsk	0.33	3.55E-14	P < 0.05
Core-top samples	0.40	7.18E-10	P < 0.05
All data	0.085	1.34E-27	P < 0.05

Supplementary Table ST8. Data table showing analysed porosity, pore density, mean pore size of *Bolivina spissa* from Gulf of Guayaquil (M77/2-59-01), Mexican Margin (MAZ-1E-04), Sea of Okhotsk (MDO1-2415), and the core-top samples – Sagami Bay (Japan), continental margin off Costa Rica (SO206-43-MUC).

Supplementary Table ST9. Data of Inter-species comparison of pore parameters and pore density vs $[NO_3^-]_{BW}$ calibration in the core-top samples.

Supplementary Table ST10. Data of bottom-water oxygen, temperature, salinity, water depth, nitrate of Peruvian OMZ, Costa Rica, and Sagami Bay with the average pore density of *B. spissa*
